# Antimicrobial Peptides Secreted From Human Cryopreserved Viable Amniotic Membrane Contribute to its Antibacterial Activity

**DOI:** 10.1038/s41598-017-13310-6

**Published:** 2017-10-20

**Authors:** Yong Mao, Tyler Hoffman, Anya Singh-Varma, Yi Duan-Arnold, Matthew Moorman, Alla Danilkovitch, Joachim Kohn

**Affiliations:** 1 New Jersey Center for Biomaterials Rutgers University 145 Bevier Rd., Piscataway, NJ 08854 United States; 2 Osiris Therapeutics, Inc, Columbia, MD 21046 United States

## Abstract

Chronic wounds remain a large problem in the field of medicine and are often associated with risk of infection and amputation. Recently, a commercially available human cryopreserved viable amniotic membrane (hCVAM) has been shown to effectively promote wound closure and reduce wound-related infections. A sprevious study indicates that hCVAM can inhibit the growth of bacteria associated with chronic wounds. In the present study, we investigated the mechanism of hCVAM antimicrobial activity. Our data demonstrate that antimicrobial activities against common pathogens in chronic wounds such as *P*.*aeruginosa*, *S*.*aureus* and Methicillin-resistant *S*.*aureus* (MRSA) are mediated via the secretion of soluble factors by viable cells in hCVAM and that these factors are proteins in nature. Further, we show that genes for antimicrobial peptides (AMPs) including human beta-defensins (HBDs) are expressed by hCVAM and that expression levels positively correlate with antimicrobial activity of hCVAM. At the protein level, our data indicate that HBD2 and HBD3 are secreted by hCVAM and directly contribute to its activity against *P*. *aeruginosa*. These data provide evidence that soluble factors including AMPs are hCVAM antimicrobial agents and are consistent with a role for AMPs in mediating antimicrobial properties of the membrane.

## Introduction

Chronic wounds are those that fail to progress through the normal repair process, and such wounds often do not respond to a standard of care and require advance wound care treatment modalities. Bacterial colonization in open wounds is a major contributor to stalled wound healing^1,2^. Measures to control bacterial colonization and growth have been shown to promote wound closure, thus are beneficial to chronic wound treatment.

Fresh human placental membranes have been used in treating wounds for more than 100 years^3,4^. However, the short shelf life limits clinical use. The development of various preservation methods allows for the long term storage of placental membranes. One of these methods uses a proprietary cryopreservation solution that retains all the components of fresh tissue, extracellular matrix, growth factors, and cells, in their native state. Studies have shown that the preservation of all components of the amnion is important for the retention of its functionality^5^. In a recent clinical study, a human cryopreserved viable amniotic membrane (hCVAM) promoted wound closure and reduced wound-related infections in treating chronic diabetic foot ulcers in comparison with the standard of care^6^. *In vitro* studies demonstrated a significant reduction in the growth of ESKAPE (*Enterococcus faecium*, *Staphylococcus aureus*, *Klebsiella pneumoniae*, *Acinetobacter baumannii*, *Pseudomonas aeruginosa or Enterobacter aerogenes*) pathogens in the presence of hCVAM, suggesting that hCVAM retains intrinisic antimicrobial properties of the amnion^7^. These properties likely contribute to the positive outcomes observed with hCVAM in clinical studies.

Endogenous antimicrobial peptides (AMPs) provide the first line of defense against microbial infections^8^. Constitutive or induced expression of AMPs occurs in many types of cells and represent one of the mechanisms of the innate immune response^9,10^. While AMPs inhibit pathogen’s growth via several pathways, the majority of the positively-charged human AMPs inhibit bacterial growth by disrupting the negatively-charged bacterial membrane^8,11^. In addition, AMPs such as human beta-defensins (HBDs), histones, and cathelicidin have also been shown to neutralize lipopolysaccharide (LPS)-induced inflammation in chronic wounds^10,12–15^.

Previous studies have demonstrated that endogenous viable cells in fresh amnion and hCVAM are the source of cytokines and immune modulators which mediate a number of functional properties of the membrane^5,16–18^. Multiple AMPs, including HBDs, elafin, secretory leucocyte protease inhibitor (SLPI), and histone H2B have been detected in fresh amniotic membranes and may protect wounds by inhibiting bacterial growth and modulating immune response^12,19–22^. The objective of this study was to investigate mechanisms of antimicrobial activity of hCVAM. Our hypothesis was that hCVAM antimicrobial activity is mediated by AMPs secreted by endogenous viable cells. A liquid culture assay was utilized to quantify the antimicrobial activity of hCVAM against *P*.*aeruginosa*, *S*.*aureus* and Methicillin-resistant *S*.*aureus* (MRSA), common pathogens in chronic wound^23^. The importance of viable cells in hCVAM was investigated by comparing of hCVAM antimicrobial activity to its devitalized counterpart. To investigate the relevance of AMPs, and HBDs, in particular, as hCVAM antimicrobial agents, we used qPCR to detect AMP gene expression in hCVAM and ELISA to measure levels of secreted AMPs. Finally, we used selective immunodepletion of HBD 2 and 3 to confirm their role in hCVAM antimicrobial activity. Results demonstrate that HBDs secreted by endogenous cells mediate hCVAM antimicrobial activity against *P*. *aeruginosa*.

## Results

### Antimicrobial activity of hCVAM is mediated by soluble factors of protein origin that secreted by tissue viable cells

**Figure 1:**
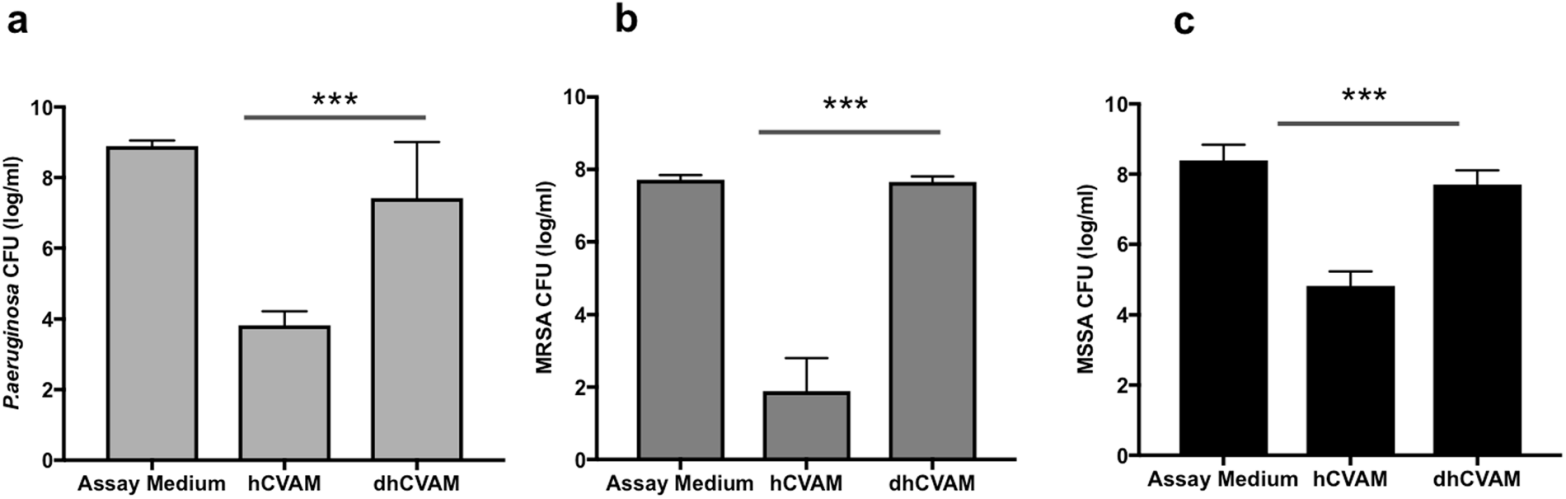
hCVAM antimicrobial activity is mediated by soluble factors secreted by tissue viable cells. Comparison of hCVAM antimicrobial activity with that from air-dried devitalized membrane (dhCVAM). hCVAM and devitalized membrane dhCVAM-derived conditioned media were generated as described in *Materials and Methods*. Effect of both conditioned media on *P*.*aeruginosa* (**a**), MRSA (**b**) or MSSA (**c**) growth was investigated. While hCVAM conditioned medium possesses significant antimicrobial properties, dhCVAM does not, demonstrating that soluble antimicrobial factors are released from tissue viable cells. Data are presented as mean ± SD of CFU in log/ml (n ≥ 3) ***p < 0.001.

Our previous work has demonstrated that hCVAM has intrinsic antimicrobial activity^7^. To determine a nature of antimicrobial factors, *P*.*aeruginosa*, MRSA, or MSSA were inoculated to hCVAM conditioned medium. As shown in Fig. 1, hCVAM conditioned medium inhibited the growth of *P*. *aeruginosa* (*a*), MRSA (*b*), and MSSA (*c*) by 5.0, 5.8, and 3.6 logs, respectively, compared to the assay medium control. This result indicates that soluble antimicrobial factors are released from hCVAM tissues in conditioned medium. Interestingly, MRSA growth was more sensitive to hCVAM condition medium than MSSA, suggesting that secreted soluble factors play a larger role in hCVAM activity of methicillin-resistant forms of *S*. *aureus* than methicillin-sensitive forms.

To demonstrate the importance of viable cells in antimicrobial activity of hCVAM, hCVAM was air-dried, a method which effectively destroys the viable cells^24^, and conditioned medium was obtained from air-dried devitalized hCVAM, and its effect on bacterial growth was tested. The data demonstrate that devitalized hCVAM (dhCVAM) did not significantly inhibit the growth of *P*. *aeruginosa*, MRSA and MSSA compared to the controls. Similar results were observed with conditioned medium derived from hCVAM, which was devitalized by a freeze/thaw hCVAM devitalization technique (Supplemental Fig. 1). These data show that the secretion of soluble antimicrobial factors is dependent on endogenous viable cells in hCVAM.

**Figure 2:**
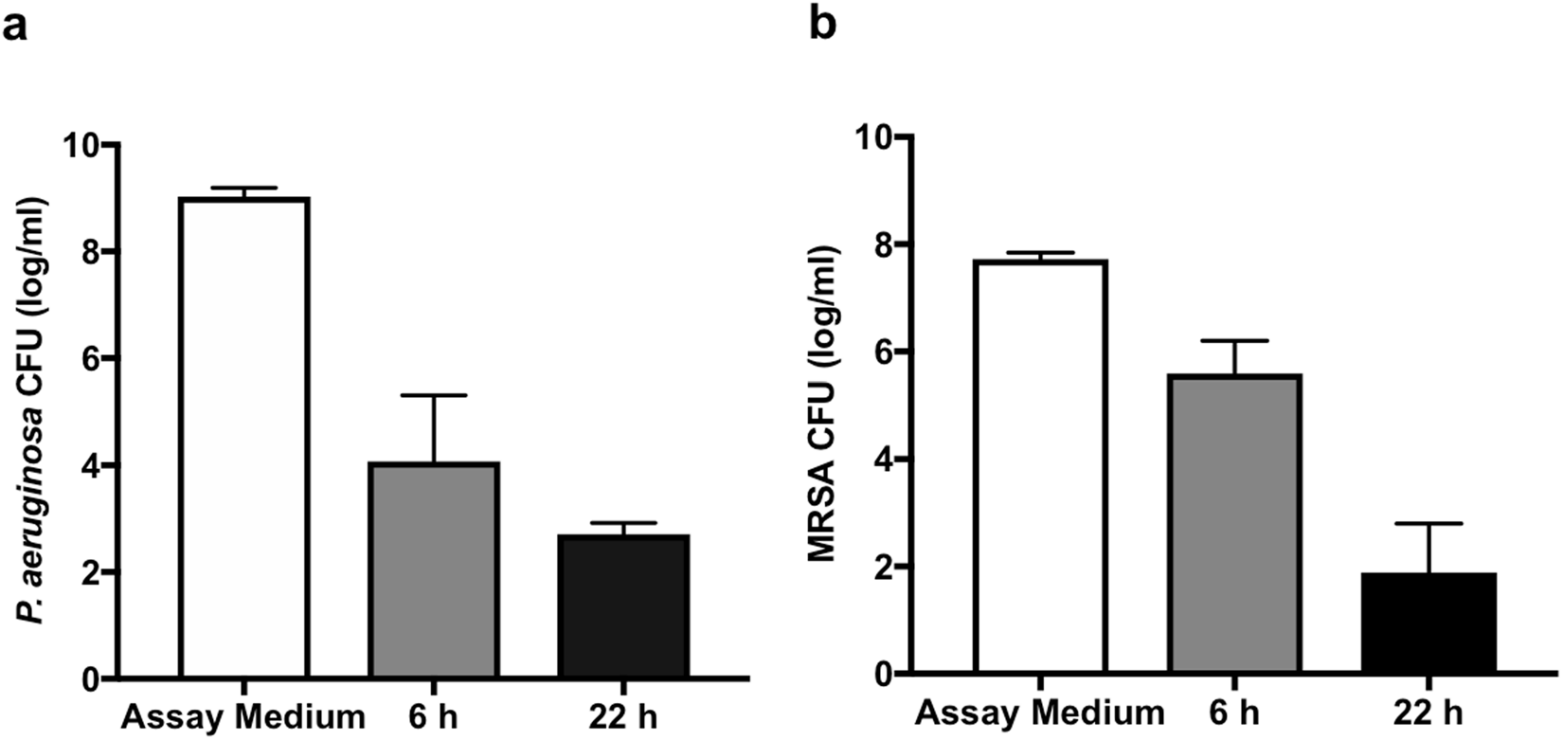
Time-dependent accumulation of antimicrobial factors in hCVAM conditioned media. hCVAM conditioned medium was collected after 6 h or 22 h in culture and assayed for antimicrobial activity against *P*. *aeruginosa* (**a**) or MRSA (**b**). For both bacteria, the data demonstrate a decrease in bacterial growth, corresponding to an increase in antimicrobial factors in the conditioned medium, over time. Data are presented as mean ± SD of CFU in log/ml (n = 3).

To investigate the time-dependence of hCVAM antimicrobial factor secretion, we compared the antimicrobial activity of hCVAM conditioned medium after 6 h and 22 h in culture (Fig. 2). The data demonstrate 6 h-conditioned medium inhibited the growth of *P*. *aeruginosa* by 5.0 logs while 22 h inhibition was 6.3 logs (Fig. 2a). Similarly, 6 h-conditioned medium inhibited MRSA growth by 2.1 logs while 22 h medium inhibition was 5.8 logs (Fig. 2b). Taken together, these data suggest that soluble hCVAM antimicrobial factors are continuously released and accumulate over the observed 22 h period.

**Figure 3:**
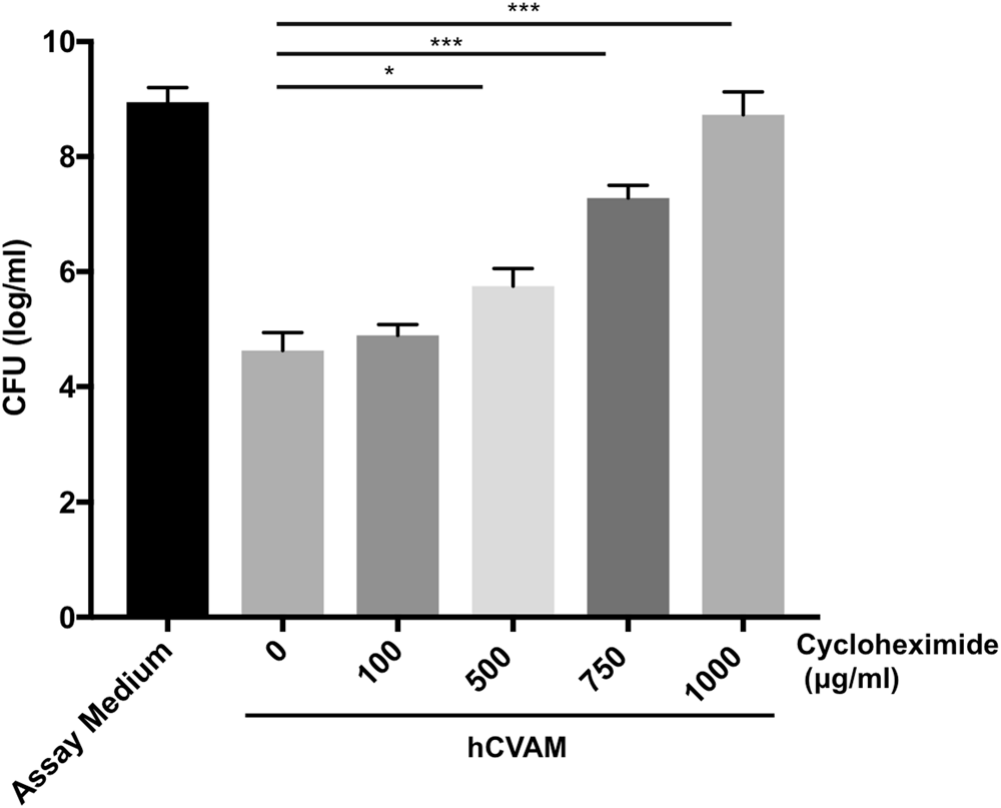
Increasing concentrations of cycloheximide results in a dose-dependent decrease in hCVAM antimicrobial activity. While the lowest concentration of cycloheximide had negligible effect, increasing concentrations of cycloheximide inhibited hCVAM antimicrobial activity. Cycloheximide at all concentrations tested did not affect *P*. *aeruginosa* growth in the absence of hCVAM (data not shown). Data are presented as mean ± SD of CFU in log/ml (n = 3). *p < 0.05 ***p < 0.001.

To determine whether soluble factors that mediate hCVAM antimicrobial activity against *P*. *aeruginosa*, are protein origin, the antimicrobial activity assays were repeated in the presence of cycloheximide, a known eukaryotic-specific inhibitor of protein synthesis^25^. Cycloheximide demonstrated a dose-dependent decrease in hCVAM antimicrobial activity (Fig. 3). At 100 µg/ml cycloheximide, the lowest concentration added, hCVAM inhibition was 4.1 logs, which was not statistically different from 4.3 logs observed for the 0 µg/ml condition. In contrast, at 1000 µg/ml cycloheximide, the highest concentration added, a significant reduction of hCVAM antimicrobial activity was observed as *P*. *aeruginosa* growth reached the assay medium control level. Taken together, these data demonstrate that the observed antimicrobial activity is dependent on protein synthesis and strongly suggests that secreted factors are proteins in nature.

### Selective antimicrobial peptides are expressed in hCVAM

**Figure 4:**
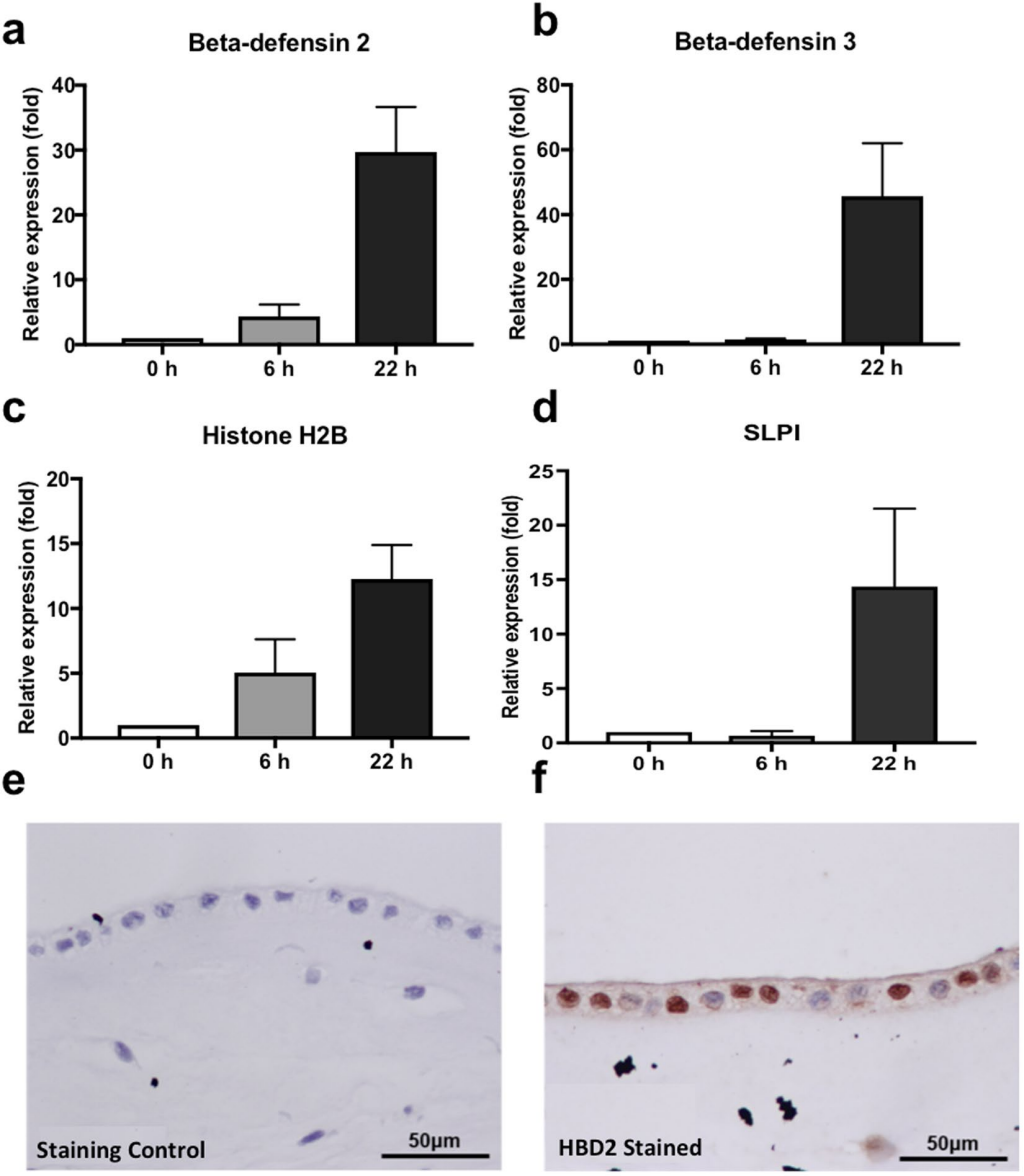
AMPs are expressed in hCVAM. (**a**,**b**) AMP gene expression in hCVAM as determined by qPCR. hCVAM tissue was processed and analyzed as described in Materials and Methods after 6 and 22 h in culture. Uncultured hCVAM (0 h) served as the control. A time-dependent increase in gene expression levels was observed for HBD2 (**a**), HBD3 (**b**), Histone H2B (**c**) and SLPI (**d**). Data are presented as mean ± SD of fold increase (n = 3). HBD2 is present in hCVAM as demonstrated by immunohistochemical staining with HBD2 antibodies: the positive brown stain seen in (**f**), but not in the negative staining control (**e**).

**Figure 5:**
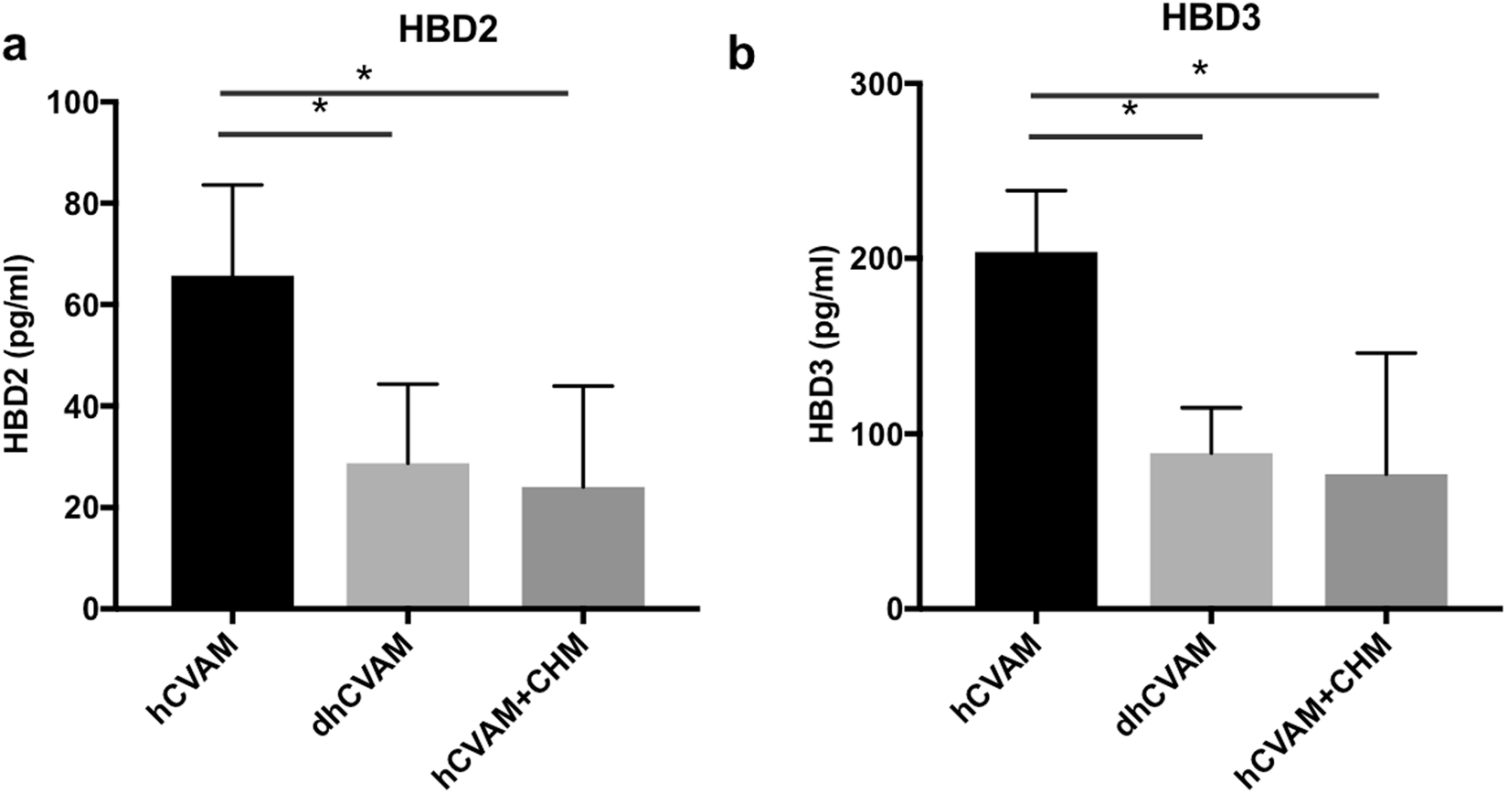
HBDs 2 and 3 proteins are present in the hCVAM conditioned medium. While HBD2 (**a**) and HBD3 (**b**) are released from hCVAM over a 24 h culture period, secretion is compromised in devitalized membrane (dhCVAM), as detected by ELISA. Blocking hCVAM protein synthesis via cycloheximide (CHM) treatment has a similar inhibitory effect as membrane devitalization. Data are presented as mean ± SD (n = 4). *p < 0.05.

Previous studies have shown that many antimicrobial peptides (AMPs), including, LL37, beta-defensins 1–3 (HBD 1–3), histone H2B, SLPI and elafin are present in human amniotic membrane^26^. To investigate the presence of these AMPs and their potential role in hCVAM antimicrobial activity, the gene expression of selected four AMPs in hCVAM was analyzed using qPCR (Fig. 4). A significant increase in gene expression levels for the 22 h time period was observed for all tested AMPs: HBD2 (Fig. 4a), HBD3 (Fig. 4b), Histone H2B (Fig. 4c), and SLPI (Fig. 4d). Interestingly, AMP gene expression profiles correlated with the observed time-dependent accumulation of antimicrobial factors in the hCVAM conditioned medium (Fig. 2). The presence of HBD2 protein in hCVAM was confirmed by immunostaining. Staining showed highest level of HBD2 in the epithelial layer of hCVAM (Fig. 5f). Taken together, these data demonstrate that selective AMP genes are expressed in cultured hCVAM and that HBD2 is present in the tissue.

### HBDs 2 and 3 are soluble factors secreted from hCVAM that contribute to its antimicrobial activity against *P*. *aeruginosa*

Performed experiments have shown that hCVAM secretes soluble factors with antimicrobial properties, and that antimicrobial peptide HBD2 and 3 genes (and HBD2 protein) are expressed in cultured hCVAM. ELISAs further confirmed the presence of HBD2 and HBD3 proteins in hCVAM conditioned medium (Fig. 5). As shown in Fig. 5a, higher levels of HBD2 were detected in hCVAM conditioned medium compared to dhCVAM, with values of 65.7 and 28.8 pg/ml, respectively. As expected, in the presence of cycloheximide HBD2 protein level was significantly decreased to 24.1 pg/ml. Similar results are shown for HBD3 (Fig. 5b), with values of 203.8, 89.0, and 77.0 pg/ml obtained for hCVAM, dhCVAM, and hCVAM in the presence of cycloheximide, respectively. These data demonstrate that both HBD2 and HBD3 AMPs are secreted by viable cells in hCVAM, and their production may require active protein synthesis.

**Figure 6:**
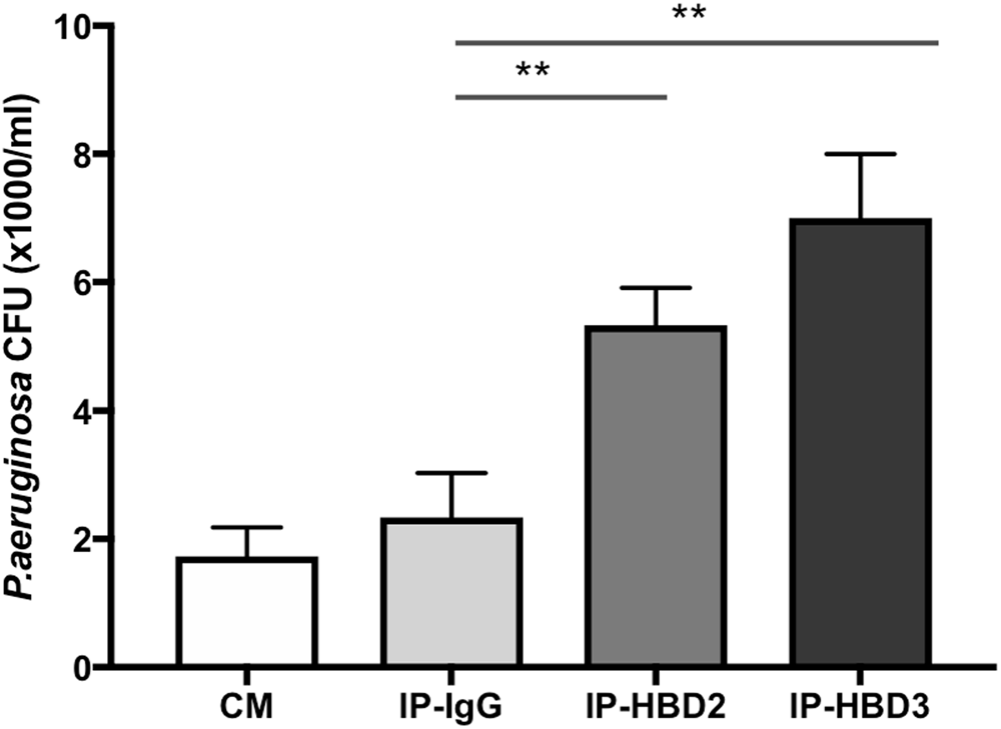
Antimicrobial peptides HBD2 and HBD3 contribute to the antimicrobial activity of hCVAM against *P*. *aeruginosa*. Depletion of HBD2 and HBD3 from hCVAM conditioned medium via immunoprecipitation (IP) with HBD2 and 3 antibodies reduced antimicrobial activity against *P*. *aeruginosa*, while an IgG control antibody did not. Control represents hCVAM 24 h conditioned medium (CM) without immunprecipitation. Data are presented as mean ± SD of CFU in 1 × 10^3^/ml (n = 3) and are a representative of four independent experiments. One-way ANOVA with a Tukey’s multiple comparisons test was performed between IP with antibodies and IP with control IgG. **p < 0.01.

To further investigate a role of these two AMPs in hCVAM antimicrobial activity, immunoprecipitation (IP) with HBD2 and HBD3 antibodies was performed with the purpose to reduce amount of HBD2 and HBD3 proteins in hCVAM conditioned medium. HBD2 and 3-depleted conditioned media were then assayed for anti-*P*.*aeruginosa* activity. In this set of experiments, rabbit-anti-mouse IgG was used as a negative control. As shown in Fig. 6, IP of HBD2 or HBD3 significantly reduced hCVAM antimicrobial activity within 1 log compared to the IgG negative control, which had no significant effect. Taken together, these data demonstrate that HBD2 and HBD3, at least in part, are responsible for hCVAM *P*. *aeruginosa*-specific antimicrobial activity.

## Discussion

Our previous study demonstrated that a human cryopreserved amniotic membrane has intrinsic antimicrobial activity against a broad spectrum of bacteria associated with chronic wounds^7^. In this study, we focused on *P*. *aeruginosa*, one of the most common pathogens in chronic wounds, and two *S*. *aureus* strains, MRSA and MSSA. We show that soluble antimicrobial agents are secreted by viable cells in hCVAM, as devitalization of the membrane shows negligible antimicrobial activity in a liquid culture assay. In addition, we show that these factors are, at least in part, proteins in nature. Furthermore, we demonstrate that selective AMP genes are expressed in hCVAM, and that they play a pivotal role in hCVAM antimicrobial activity against *P*. *aeruginosa*.

Both freeze-thaw and air-dry methods effectively devitalized hCVAM. Importantly, devitalization of hCVAM had a negative impact on antimicrobial activity and secretion of both HBD2 and HBD3 from the membrane. These data strongly support the critical role of viable cells in hCVAM antimicrobial activity.

Reduction of bacterial growth in our liquid culture assay may have clinical significance by improving the wound environment. Reducing bacterial growth may decrease bacterial load to a level that does not inhibit wound healing^27^. It has been shown that even a decrease of one log10 (90%) bacterial load (3 × 10^6^ staphylococci/ml) led to clinical improvements in pneumonia patients^28^. Studies have indicated that inhibition of bacterial growth rate may alter the adherence of bacteria to epithelial cells and increase bacterial susceptibility to host immune defense^29^. The presence of AMPs may enhance the activities of antibiotics through synergistic effects and make the combination treatment more potent^30^.

In this study, we demonstrate a positive correlation between hCVAM antimicrobial activity and gene expression of AMPs HBDs 2 and 3 and protein levels. Using ELISA, we quantified HBD2 and HBD3 proteins in hCVAM conditioned medium. In addition, while secreted protein levels of both AMPs were reduced, the low level was detected in both devitalized membrane (dhCVAM) and cycloheximide conditions (Fig. 5). This result suggests that although viable cells are the main source of secreted antimicrobial peptides, AMPs are present at detectable levels in the tissue extracellular matrix. A role for HBDs 2 and 3 as hCVAM antimicrobial agents against *P*. *aeruginosa* was confirmed by selective removal of each via immunoprecipitation from conditioned medium (Fig. 6).

Previous studies have shown that the methicillin-resistant strain of *S*.*aureus*, MRSA, is more sensitive to antimicrobial peptides, such as beta-defensins, compared to the methicillin-sensitive strain, MSSA^31^. Here, we show that MRSA is more sensitive to hCVAM conditioned medium than MSSA (Fig. 1). These data suggest that AMPs secreted by hCVAM are more effective antimicrobial agents against MRSA than MSSA. This question as well as hCVAM mechanisms of antimicrobial activity against other ESKAPE bacteria besides *P*. *aeruginosa* will be addressed in future studies.

In summary, we demonstrate that soluble antimicrobial factors secreted by viable cells in hCVAM play a key role in hCVAM antimicrobial activity, and beta-defensins 2 and 3 are important contributors to hCVAM antimicrobial activity against *P*. *aeruginosa*.

## Methods

### Preparation of hCVAM samples and conditioned medium

hCVAM samples derived from different donors were prepared as previously described^17^. Human term placenta tissues were procured and processed at Osiris Therapeutics, Inc. (Columbia, MD) following its proprietary manufacturing procedure. The tissue procurement and ethics statement were provided by The National Disease Research Interchange (Philadelphia, PA) and Cord Blood America, Inc. (Las Vegas, NV) from eligible donors after obtaining written informed consent.

Prior to experiments, hCVAM tissues stored at −80 °C were thawed in a room temperature water bath for 3–5 min. hCVAM tissue was removed from a cryobag, placed in a sterile basin and washed with sterile PBS. To generate conditioned medium. hCVAM tissue was incubated in a sterile 50 ml conical tube containing assay medium (1 ml per 4 cm^2^ of hCVAM) consisting of Dubelco’s Modified Eagle Medium (DMEM), (Invitrogen) and 10% fetal bovine serum (FBS) (Atlanta Biologicals), with shaking at 37 °C, in a cell incubator set for 5% CO_2_ and 95% humidity. Conditioned medium was collected at 24 h for single time point experiments and at 6 and 22 h for multiple time point experiments, unless otherwise reported. Following collection, conditioned medium was either used immediately or stored at −80 °C.

### Bacterial culture and preparation of inoculums

Clinical isolates of bacterial strains *Pseudomonas aeruginosa* (*P*. *aeruginosa*; ATCC^®^ 15692™), *methicillin-resistant Staphylococcus aureus* (*S*. *aureus*) (MRSA; ATCC^®^ BAA-1720) and methicillin-sensitive *S*. *aureus* (MSSA; *A*TCC^®^ 25923™), were maintained following instructions provided by the supplier. Preparation of bacterial inoculum was performed as described previously^7^. Briefly, bacteria were cultured in tryptic soy broth at 37 °C with shaking until the absorbance optical densities measured in the range of 0.2 to 0.6 at a wavelength of 600 nm. The number of colony forming units (CFUs) for each strain was estimated based on an OD_600_ = 1.0, which corresponds to 10^9^ CFU/ml. To prepare the inoculum for hCVAM antimicrobial assays, the bacterial stock solution was diluted with either assay medium or conditioned medium to approximately 100 CFU/ml of bacteria. For each experiment, the actual CFU of each inoculum was determined by preparing serial dilutions and plated onto tryptic soy broth agar plates.

### Antimicrobial Activity Assays

100 CFU of *P*. *aeruginosa* or *S*. *aureus* were cultured with 1 ml of assay medium or conditioned medium and incubated at 37 °C with shaking for 24 h. Serial dilutions were then prepared of each culture and plated onto tryptic soy broth agar plates. CFUs were counted after overnight incubation at 37 °C.

### Devitalization of hCVAM

Devitalization of hCVAM was done by an “air-dry” method. Briefly, the membrane was thawed, washed in PBS, and transferred to a sterile petri dish. hCVAM was air-dried under sterile conditions for 3–5 h. The membrane was then rehydrated in PBS for 30–60 min before use.

Devitalization of hCVAM was also done using a “freeze-thaw” method. The membrane was thawed, washed in sterile water, and transferred to a petri dish in a −80 °C freezer for 25 min. Frozen hCVAM was then thawed in water at room temperature for 25 min. This freeze/thaw cycle was repeated once.

The lack of viable cells was confirmed by staining of devitalized hCVAM with LIVE/DEAD viability/cytotoxicity kit (Invitrogen).

### Inhibition of hCVAM protein synthesis via cycloheximide treatment

hCVAM was cultured in assay medium for 24 h in the presence of cycloheximide at 100, 500, 750 or 1000 µg/ml. Conditioned medium was collected and used in a *P*. *aeruginosa* antimicrobial activity assay. Assay medium containing listed above concentrations of cycloheximide served as the controls for these experiments.

### Antimicrobial peptide gene expression in hCVAM using q-PCR

To isolate RNA for antimicrobial peptide (AMP) quantitative PCR (q-PCR) experiments, Freshly thawed (0 h) or cultured for 6 and 24 h in assay medium hCVAM tissues were used. Conditioned medium was collected and stored at −80 °C for future testing for AMPs while the membrane was transferred to a fresh tube containing PBS. After a single PBS rinse, the tissue was dried using a kimwipe and flash frozen in liquid nitrogen for 2 min. The frozen hCVAM tissue was pulverized using a mortar and pestle and resuspended in 1 ml of RNA lysis buffer (Promega). Samples were transferred to a 5 ml Eppendorf tube and further homogenized using an Ultra-Turrax (IKA T10) homogenizer for 3 15-second cycles followed by 1 min of cooling on wet ice. The lysates were centrifuged at 12000 rpm for 5 min and supernatants were used for RNA extraction/purification using SV 96 Total RNA Isolation System (Promega). RNA concentration and purity was measured using Nanodrop2000 (Thermo Scientific). The cDNA synthesis and qPCR analysis were performed as described^32^. The primers used for qPCR include Quanti-tech GAPDH (QT01192646), Beta-defensin 2 (QT00204617), Beta Defensin 3 (QT01529535), Secretory Lyphocyte protease inhibitor (SLPI) (QT00236117), Histone H2B (QT00199941) (Qiagen). Following each run, a second derivative analysis was performed using raw data to determine the mean Cp for each sample. GAPDH expression served as an internal control. Relative mRNA expression was determined by Pfaffl analysis (EΔCp target/EΔCp reference) in which primer efficiency E = 10^(−1/slope) and ΔCp = mean Cp of sample - mean Cp of the experimental control. The relative expression in hCVAM tissues cultured 6 or 24 hr in the assay medium was normalized to the expression in freshly thawed hCVAM (0 h).

### Immunohistochemical staining of human beta-defensin 2 in hCVAM

Following a 22-h incubation in assay medium, hCVAM tissue was isolated, rinsed twice with PBS, and fixed in 4% paraformaldehyde for 24 h. After fixation, the membrane was washed in PBS and stored at 4 °C in 70% ethanol. Samples were sections and stained by Histoserv, Inc. Sections were stained with anti-human beta-defensin 2 (HBD2) goat antibodies (Santa Cruz) as previously described^26^. Briefly, sectioned were stained with primary antibody followed by biotinylated secondary rabbit anti-goat IgG (Vector Laboratories). Diaminobenzidine (Sigma) was used to visualize positively stained cells. Sections stained without the primary antibody served as the negative staining control.

### Detection of human beta defensins using ELISA

hCVAM or devitalized hCVAM were cultured in assay medium for 24 h. Conditioned medium after 24 h incubation was collected and analyzed for HBD2 and human beta-defensin 3 (HBD3) using ELISA (Human BD-2 Mini ABTS ELISA kit and Human BD-3 Mini ABTS ELISA kit) according to the manufacturer’s instructions (PeproTech).

### Immunoprecipitation

Selective depletion of HBD2 and HBD3 from hCVAM conditioned medium was accomplished by immunoprecipitation (IP). Conditioned medium was collected from multiple lots of hCVAM cultured for 24 h in assay medium. Rabbit polyclonal antibodies against HBD2 (Santa Cruz sc-20798) or HBD3 (Santa Cruz sc-30115) or a non-specific rabbit IgG (control) (ThermoFisher Scientific #31555) was added to a 1 ml sample of conditioned medium at a final concentration of 1 μg/ml and incubated with mixing overnight at 4 °C. A 100-µl slurry of Protein A/G agarose plus resin (ThermoFisher Scientific #20421) was washed twice with 1 ml of PBS and mixed with each condition medium sample for 2 h at room temperature. Mixtures were then centrifuged at 2000 rpm for 3 min and resulting supernatants were transferred to individual bacterial culture tubes. 100 CFU of *P*.*aeruginosa* was added to each culture tube and incubated with shaking at 37 °C. After 24 h, CFUs for each culture was quantified.

### Statistical analysis

Each independent experiment contained 3 or more biological repeat samples (n ≥ 3), and data is presented as the mean ± standard deviation. Results shown are a representative of at least two independent experiments. One-way ANOVA with a Tukey’s multiple comparisons test was performed to determine statistical significance. Differences were considered significant at a p value of < 0.05.

## Electronic supplementary material

Supplementary Information
