# Supplementary material for: Antimicrobial Peptides Secreted From Human Cryopreserved Viable Amniotic Membrane Contribute to its Antibacterial Activity
Source: Sci Rep. 2017 Oct 20;7:13722. doi: 10.1038/s41598-017-13310-6 (PMC5651856; doi:10.1038/s41598-017-13310-6)
Supplement: Supplementary file 1 — Supplementary Information [file 41598_2017_13310_MOESM1_ESM.docx]

**Supplementary Information**

**Antimicrobial Peptides Secreted From Human Cryopreserved Viable Amniotic Membrane Contribute to its Antibacterial Activity**

Yong Mao1, Tyler Hoffman1, Anya Singh-Varma1, Yi Duan-Arnold2, Matthew Moorman2, Alla Danilkovitch2 and Joachim Kohn1

1. New Jersey Center for Biomaterials Rutgers University

145 Bevier Rd., Piscataway, NJ 08854

1. Osiris Therapeutics, Inc. Columbia, MD 21046

Corresponding author: Joachim Kohn Address: 145 Bevier Rd., Piscataway, NJ 08854

E-mail: [kohn@dls.rutgers.edu](mailto:kohn@dls.rutgers.edu) Telephone: (848) 445 9611

***P.aeruginosa***

# 10

**8**

**CFU (log/ml)**

**6**

**4**

**2**

**0**

**Medium ctrl hCVAM dhCVAM**

Figure S1: **Devitalization by freeze/thawing abolishes hCVAM antimicrobial activity.** Comparison of hCVAM antimicrobial activity with that from devitalized membrane (dhCVAM) using a freeze/thaw technique. This technique and the preparation of conditioned media are described in *Materials and Methods*. Antimicrobial activity against *P.aeruginosa* was observed for the conditioned medium derived only from the mebrane containing viable cells. This result demonstrates that viable cells in hCVAM are responsible for secretion of soluble antimicrobial factors. Data are presented as mean ± SD of CFU in log/ml (n=3).
